# Unveiling the mechanism of ultrasound-assisted phenolic extraction from *Psidium cattleianum* leaves: Kinetic, mass transfer, and thermodynamic insights^☆^

**DOI:** 10.1016/j.ultsonch.2025.107675

**Published:** 2025-11-11

**Authors:** Hoang Duy Huynh, Parushi Nargotra, Hui-Min David Wang, Yung-Hsiang Tsai, Chien-Chih Chiu, Chwen-Jen Shieh, Yung-Chuan Liu, Chia-Hung Kuo

**Affiliations:** a Department of Seafood Science, National Kaohsiung University of Science and Technology, Kaohsiung 811, Taiwan; b Institute of Aquatic Science and Technology, National Kaohsiung University of Science and Technology, Kaohsiung 811, Taiwan; c Faculty of Applied Technology, Yersin University of Dalat, Dalat 670000, Vietnam; d Graduate Institute of Biomedical Engineering, National Chung Hsing University, Taichung 402, Taiwan; e Department of Biotechnology, Kaohsiung Medical University, Kaohsiung 807, Taiwan; f Biotechnology Center, National Chung Hsing University, Taichung 402, Taiwan; g Department of Chemical Engineering, National Chung Hsing University, Taichung 402, Taiwan

**Keywords:** Strawberry guava leaf, Ultrasonication, Bioactive compounds, Pseudo-second-order model, Diffusion, Thermodynamic state

## Abstract

Strawberry guava leaves (*Psidium cattleianum*) are an attractive phenolic-rich byproduct for food and pharmaceutical production, with potential benefits for human health due to their exceptional antioxidant activity among plant-based resources. However, there are no systematic studies on sonochemical phenolic extraction from this material. Therefore, the goal of this study was to unveil the mechanism of ultrasound-assisted extraction (UAE) for phenolic recovery from *Psidium cattleianum* leaves. Pseudo-second-order kinetic modeling, Biot number, and thermodynamic analysis were introduced to describe the extraction mechanisms and then kinetic parameters such as saturation concentration (*C_s_*), extraction rate (*k)*, diffusion coefficient (*D_eff_*), and mass transfer coefficient (*K_T_*) compared with conventional shaking extraction (CSE) to establish the process intensification benefits. The result of pseudo-second-order kinetic analysis revealed a biphasic extraction mechanism consisting of initial rapid release, followed by diffusion-controlled mass transfer, with internal mass transfer identified as the rate-limiting step (*Bi* > 50). The UAE process was spontaneous (ΔG^o^ < 0), endothermic (ΔH^o^ = 3.8151 kJ/mol), and entropy-driven (ΔS^o^ > 0). The presence of ultrasound technology enabled UAE to demonstrate superior performance compared to CSE, which significantly increased *C_s_*, *k, D_e_* and *K_T_*. Taken together, the integration of kinetic and thermodynamic analyses offers a robust framework for understanding sonochemical processes and fundamental insights into the nature of UAE from *Psidium cattleianum* leaves, thereby overcoming scale-up challenges and facilitating industrial applications.

## Highlights

• UAE was efficient in extracting bioactive compounds from *P. cattleianum* leaves.
• Pseudo-second-order modeling precisely fits extraction kinetics (R^2^ > 0.99).
• Biphasic UAE kinetics identified internal mass transfer as the rate-limiting step.
• UAE exhibited spontaneous, endothermic, and entropy-driven thermodynamics.

## Graphical abstract

**Figure:**
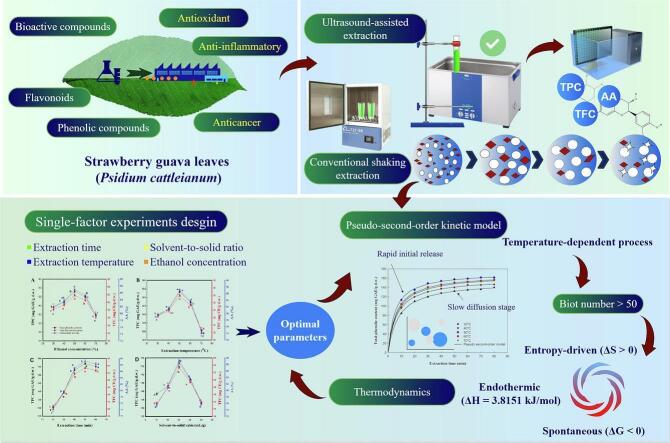

## 1 Introduction

*Psidium cattleianum*, commonly called strawberry guava, is an exotic species belonging to the Myrtaceae family [1]. As a slow-growing, fruit-bearing tree, it is native to southeastern Brazil but is widely introduced to tropical and subtropical regions around the world, such as Hawaii, the Caribbean Islands, and many Asian countries [2]. Unfortunately, the presence in Asia is relatively rare, and most wild specimens are recognized, cultivated, processed, and consumed only locally [3]. Nevertheless, being a part of the guava family, strawberry guava itself and its byproducts hold a considerable potent source of bioactive compounds (BACs), including phenolic acids, flavonoids, tannins, phenol lipids, and carbohydrates, which are mostly obtained from the leaf extract, even with greater diversity than other well-known leaves such as mate or tea [4]. Phenolic compounds from strawberry guava leaf (SGL) extract are critical components in secondary metabolites of this plant and possess the ability to be antioxidant [2,5], anti-inflammatory, antibacterial [4], antiproliferative, and restraining various forms of malignant cancer [6]. Despite these promising bioactivities, current studies on native species of strawberry guava, typically from Brazil, have focused on the study of fruits, while studies on the utilization of SGL byproducts are still very limited, especially when it comes to systematic, scientific, or technological research.

Ultrasound-assisted extraction (UAE) has been emerging as a green technology for the extraction of polyphenols from plant matrices [7]. By overcoming the limitation of conventional soaking extraction (CSE), many interesting advantages are shared by the UAE method, including high extraction yield, low energy consumption, decreased extraction time, reduced environmental impact, and preservation of the targeted compounds with minimal degradation [8]. Additionally, for scaling up industrial production, this process represents one of the simplest and most affordable options. To date, ultrasound treatment is widely employed for the development of various value-added products that contribute toward achieving Sustainable Development Goals [9]. The core principle behind the operation of UAE is cavitation, which is generated by high-intensity ultrasound typically within a low frequency ranging from 20 kHz to 100 kHz [10]. More specifically, during UAE, the formation, growth, and then collapse of cavitation bubbles lead to the disruption of cell walls and enhance the mass transfer rate as well, thereby improving the penetration of the solvent into the solid matrix [11]. It is important to note that in extraction processes, the useful product is the extractable solute, and the presence of solutes affects the application of ultrasound in a number of ways. Indeed, the UAE’s effectiveness depends on many variables, including ultrasonic power, frequency, solvent type and concentration, solvent-to-solid ratio, extraction time, and temperature [8,12]. Therefore, understanding the influence of these variables on extraction procedures and yields of phenolic compounds is crucial for simulating and designing UAE processes.

To achieve the technological transfer from laboratory to industrial scale, the nature of solid–liquid extraction must be investigated in detail. For this, based on molecular transport principles, the analysis and design of extraction operations require knowledge in three main areas: equilibrium, energy and material balance, and kinetics [13]. As a crucial component for scale-up, various kinetic models are employed, with the pseudo-second-order kinetic model being a widely employed mathematical framework for analyzing the kinetics of solid–liquid extraction processes due to its ability to precisely capture these phenomena [14,15]. Dealing with difference equations, this model aims to calculate key parameters such as extraction rate, diffusion coefficient, and mass transfer coefficient to understand mass transfer mechanisms, optimization, and scalability. Thermodynamic parameters such as enthalpy (ΔH^o^), entropy (ΔS^o^), and Gibbs free energy (ΔG^o^) offer insights into understanding the mass and heat transfer rates, thus supporting their optimization and control according to specific extraction conditions. To date, no study has been reported on the kinetics and thermodynamics of green extraction of phenolic compounds from SGL using ultrasonication with ethanol as the liquid solvent.

This study aims to investigate the UAE technique for recovering phenolic compounds from SGL under varying parameters, including ethanol–water mixture concentration, extraction time, extraction temperature, and solvent-to-solid ratio. The effects of these extraction conditions will be evaluated through single-factor experimental design. Subsequently, the pseudo-second-order kinetic models will be employed to calculate the diffusion and mass transfer coefficients, the Biot number, and thermodynamic parameters. These kinetic parameters from UAE will then be compared with CSE, to support the efficiency of UAE for sustainable recovery of BACs from SGL byproducts for applications in food and bio-based products.

## 2 Materials and methods

### 2.1 Materials

The strawberry guava leaves (*Psidium cattleianum*) were harvested manually from veteran strawberry guava trees at Cau Dat farm (Lam Dong, Vietnam). During harvesting, the leaves without damage were selected at a consistent level of maturity. The fresh leaves were washed with tap followed by deionized water. Afterwards, the cleaned leaves were dried in a hot air dryer at 45 ± 2 °C for 8 h. Finally, the dry leaves were ground into fine powder, passed through a 60-mesh sieve, packed in a sealed aluminum bag, and stored at −18 °C for subsequent analysis. Folin-Ciocalteu phenol reagent was procured from Sigma-Aldrich (St. Louis, MO, USA). 2,2-diphenyl-1-picrylhydrazyl (DPPH, 95 %) was received from Alfa Aesar (Ward Hill, MA). Gallic acid (98 %) was purchased from Combi-Blocks (San Diego, CA). Catechin hydrate (97 %) was acquired from TCI (Tokyo, Japan). Other chemicals such as sodium carbonate (99.7 %), sodium hydroxide (99.5 %), sodium nitrite (99.8 %), and aluminum chloride (99.5 %) were supplied at Showa (Tokyo, Japan). All chemicals and reagents used in this study were of analytical grade.

### 2.2 Ultrasound-assisted extraction of BACs from SGL

The UAE of bioactive components from SGL was carried out in an ultrasonic bath (Elmasonic P 70H, Elma, Siegen, Germany) under a fixed ultrasound frequency of 37 kHz and an amplitude of 60 %. Ethanol solution was used as a liquid solvent in the extraction process for recovering targeted compounds. In practice, by carefully weighing 4 g of dried SGL, the sample was combined with ethanol-water solution in a 250 mL beaker. Key parameters for the process of UAE, including ethanol concentration, extraction temperature, extraction time, and solvent-to-solid ratio, were derived from the experimental design. The extract was then centrifuged at a relative centrifugal force (RCF) of 4354 × g for 10 min and passed through Whatman No. 1 filter paper. Then, the clear supernatant liquid was concentrated using a rotary evaporator at 50 °C before being dried with a freezer dryer at −46 °C and 16 Pa. Finally, the freeze-drying extracts were stored at −18 °C for future experiments.

### 2.3 Single-factor experiment design

The single-factor experimental design was conducted to examine the effects of single factors in UAE parameters, specifically ethanol concentration (30–70 %), extraction temperature (30–70 °C), extraction time (20–60 min), and solvent-to-solid ratio (10–30 mL/g) on the total phenolic content (TPC), total flavonoid content (TFC), and antioxidant activity (AA). The single-factor design was carried out with twenty experimental runs, which aimed to provide a comprehensive insight into how each factor contributes to the UAE process optimization. All the runs in this design were conducted in triplicate; results were expressed as the mean value ± standard deviation.

### 2.4 Quality analysis of SGL extract

#### 2.4.1 Total phenolic content

The total phenolic content (TPC) of extracts was determined using the Folin-Ciocalteu spectrophotometric method with minor modifications [16]. Using gallic acid as a phenolic standard compound, the standard curve was prepared with concentrations ranging from 200 to 1000 mg/L. For sample analysis, 100 µL of the diluted extract was mixed with 200 µL of 10 % Folin-Ciocalteu reagent in a 2 mL microtube, vortexed, and then kept at room temperature (25 ± 2 °C) for 5 min. Subsequently, 800 µL of 7.5 % (w/v) Na_2_CO_3_ solution was added to neutralize the reaction, and the mixture was incubated for an additional 2 h at the same temperature. The absorbance of both sample and blank solutions was measured at 765 nm using a UV–visible spectrophotometer (UV-1280, Shimadzu, Japan). The TPC results, mg gallic acid equivalents (GAE) per g of sample in dried weight, were calculated by Eq. (1).

(1) $$Total\;phenolic\;content\;\left(mg\;GAE/g\;d.w.\right)=\;\frac{C\times V\times D_{f}}{W}$$

where, C is the concentration of gallic acid from the standard curve (mg/L), V indicates the volume of extract (L), *D_f_* = 20 denotes the dilution factor, and W is the dry weight of the sample (g).

#### 2.4.2 Total flavonoid content

As per the method by Wu et al. (2020) [17], the total flavonoid content (TFC) of extracts was measured using the aluminum chloride colorimetric method with minor modifications. The standard curve of catechin as a flavonoid standard compound was prepared with concentrations ranging from 60 to 140 mg/L. For sample analysis, a 100 μL diluted extract was mixed with 400 μL of 80 % ethanol solution and then combined with 30 μL of 5 % (w/v) NaNO_2_. The obtained mixture was incubated for a time of 6 min at room temperature (25 ± 2 °C). Following this incubation, 30 μL of 10 % w/v AlCl_3_ solution was added, and then it continued to be kept for 6 min under the same condition. Then, 200 μL of 1 M NaOH was incorporated into the mixture, and the volume was adjusted to 1 mL with 240 μL of 80 % (v/v) ethanol-water solution. The absorbance of both sample and blank solutions (500 μL each) was measured at 510 nm using a UV–visible spectrophotometer (UV-1280, Shimadzu, Japan). The TFC results, mg catechin equivalents (CE) per gram in dried weight, were calculated by Eq. (2)

(2) $$Total\;flavonoid\;content\;\left(mg\;CE/g\;d.w.\right)=\;\frac{C\times V\times D_{f}}{W}$$

where, C is the catechin concentration determined from the standard curve (mg/L), V is the volume of original extract (L), *D_f_* = 40 denotes the dilution factor, and W is the dry weight of the sample (g).

#### 2.4.3 Antioxidant activity

The antioxidant activity (AA) of the extract was measured using the 2,2-diphenyl-1-picrylhydrazyl (DPPH) free radical scavenging assay, according to the method described by Tran et al. (2024) [18]. First, a 120 μM DPPH stock solution was prepared by dissolving 0.0047 g of DPPH powder in 100 mL of analytical-grade ethanol. The solution was stored at 4 °C and used within 24 h to ensure stability. For each measurement, 30 μL of extract sample was mixed with 470 μL of 120 μM DPPH solution and then incubated in darkness for 30 min at room temperature (25 ± 2 °C). The absorbance of both the reaction mixture and the blank sample (80 % v/v ethanol concentration) was measured at 517 nm using a UV–visible spectrophotometer (UV-1280, Shimadzu, Japan). The AA was calculated by the percentage inhibition using Eq. (3).

(3) $$\mathrm{Antioxidant}\;\mathrm{acitivity}\left(\%\right)=\left(\frac{A_{\mathrm{blank}}-A_{\mathrm{sample}}}{A_{\mathrm{blank}}}\right)\times 100$$

where, $A_{\mathrm{blank}}$ is the absorbance of the blank control, and $A_{\mathrm{sample}}$ is the absorbance of the test sample.

### 2.5 Kinetic model for evaluating UAE process

#### 2.5.1 Pseudo-second-order rate constant and saturation concentration

The model begins by finding the rate of dissolution in the liquid phase over time. More specifically, the dissolution rate is proportional to the square of the difference between the saturation concentration (*C_s_*) and the concentration at a given time (*C_t_*), as expressed in Eq. (4).

(4) $$\frac{dC_{t}}{\mathrm{dt}}=k{\left(C_{s}-C_{t}\right)}^{2}$$

Where, $C_{t}$ (mg GAE g^−1^) represents the concentration of phenolic compounds derived from the extract at time *t* (min), *C_s_* (mg GAE g^−1^) is the saturation concentration at equilibrium, and *k* (g mg^−1^ min^−1^) is the second-order rate constant. Next, for evaluation of $C_{t}$, the differential equation is solved subject to the boundary conditions: $C_{t}$= $C_{0}$ at *t* = 0, and $C_{t}=C_{t}$ at *t* = *t*. Then integrate Eq. (4), which yields a linear form as follows:

(5) $$C_{t}=\;\frac{C_{s}^{2}.\;k.t}{1+\;C_{s}.k.t}$$

And linearized form:

(6) $$\frac{t}{C_{t}}=\;\frac{1}{kC_{s}^{2}}+\frac{t}{C_{s}}=\frac{1}{h}+\frac{t}{C_{s}}$$

where *h =* $kC_{s}^{2}$ (mg g^−1^ min^−1^) is the initial extraction rate. Alternatively:

(7) $$C_{t}=\;\frac{t}{1/h+(t/C_{s})\;}$$

Hence the temperature dependence of k follows the Arrhenius equation. Thus

(8) $$k=\;k_{0}\exp\left(\frac{-E_{a}}{\mathrm{RT}}\right)$$

where $k_{0}$ (g mg^−1^ min^−1^) denotes the pre-exponential factor, $E_{a}$ (J mol^−1^) is the activation energy, R = 8.314 J mol^−1^K^−1^ is the gas constant, and *T* (K) is the absolute temperature. Linearized:

(9) $$lnk=\;{lnk}_{0}+\left(\frac{-E_{a}}{\mathrm{RT}}\right)\frac{1}{T}$$

To calculate key kinetic parameters of the model, experimental data for concentration *C_t_* at various extraction times (*t* = 10–80 min) are collected, and a plot of *t*/*C_t_* versus *t* is generated from Eq. (6). The slope of this linear plot provides 1/*C_s_*, allowing calculation of the saturation concentration (*C_s_*). The intercept of the linear plot provides 1/(*k* × $C_{s}^{2}$), or 1/*h*, from which the pseudo-second-order rate constant (*k*) and the initial extraction rate (*h*) can be determined. The *k* values were determined at 30 to 70 °C from Eq. (9), plotting ln*k* vs. *1/T* to estimate precisely *E_a_* and $k_{0\;}$.

#### 2.5.2 Diffusion coefficient

The diffusion coefficient ($D_{e}$, m^2^ s^−1^) characterizes the mass transfer of phenolic compounds inside the solid matrix from SGL powder [19]. Assumed initial conditions are the following properties:

(i) Particles are spherical with a mean diameter of 0.25 mm (radius *R* = 1.25 × 10^−4^ m).
(ii) Internal diffusivity remains constant within the particle.
(iii) Initial concentration of phenolic compounds inside the particle is uniform (*C_0_*). Boundary conditions.

*C* (*r*, 0) = *C_0_* for *t* = 0 and *r* ≥ 0 (10).

${\left(\frac{\partial C}{\partial t}\right|}_{t=0}=0$ for *t* > 0 and *r* = 0 (11).

*C* (*R*, *t*) *=* $C_{S}$*= C*_∞_ for *t* > 0 and *r = R* (12).

Here, *C_s_* is the equilibrium concentration at the surface, assumed equal to the maximum extractable concentration (*C*_∞_).

(iv) Under ultrasound treatment does not significantly alter the particle structure or $D_{e}$.

The coefficient of diffusion is governed by Fick’s second law of radial diffusion, based on Crank’s model [8], so we have

(13) $$\frac{\partial C}{\partial t}=\;D_{e}\left(\frac{\partial^{2}C}{\partial^{2}r}+\frac{2}{r}\frac{\partial C}{\partial r}\right)$$

Where, *C* is the concentration of phenolic compounds (mg GAE g^−1^) at a given extraction time (*t*, min). To convert Eq. (13) through the method of separation of variables to a differential equation, we apply Crank’s solution for radial diffusion in a sphere, which describes the average unaccomplished extraction yield ratio (*y*) in the volume of the SGL undertaking boundary conditions, as written below:

(14) $$y\left(r,t\right)=\;\frac{6}{\pi^{2}}\sum_{n=1}^{\infty }\frac{{\left(-1\right)}^{2(n+1)}}{n^{2}}\left\{\exp\left[-{\left(\frac{n\pi }{R}\right)}^{2}D_{e}t\right]\right\}$$

where

(15) $$y\left(r,t\right)=\frac{C_{\infty }-C\left(r,t\right)}{C_{\infty }-C_{o}}$$

With minor adjustments, the coefficient of diffusion was determined following Nicolin et al. (2016) [20] under 1, 10, 20 terms. R software (Version 4.5.2, R Core Team, 2025) was used to calculate the parameter estimation of $D_{e}$ by fitting Eq. (14) to the experimental kinetic data y(r,t) through nonlinear least-squares regression with the “nls” function. The “port” algorithm was specified to allow for the imposition of a critical physical constraint, setting the lower bound for the diffusion coefficient to $D_{e}$ ≥ 0. To precisely quantify the uncertainty of the $D_{e}$ estimates, a non-parametric bootstrap analysis was conducted in this study. Following the methodology established by Davison and Hinkley (1997) [21], we executed a residual resampling bootstrap algorithm with the subsequent steps:

(i) Reparameterization: ${k=D}_{e}/R^{2}$;
(ii) Fit initial model *y = f (t, k)* using the robust Levenberg-Marquardt algorithm “*nls.lm*”, and then obtain the corresponding vector of centered residuals, ${∊}^{\prime }=∊-\overset{¯}{∊}$;
(iii) Generate bootstrap sample (y*) by adding resampled residuals to the fitted values;
(iv) Obtain bootstrap estimate (k*) from each pseudo-dataset;
(v) Repeat step (iii) and (iv) for 2000 iterations;
(vi) Transform to $D_{e}^{*}$ = k * R^2^ for final bootstrap distribution.

Based on the final $D_{e}^{*}$ distribution, statistical analyses, including bootstrap standard errors and 95 % bootstrap confidence intervals for $D_{e}$, were computed.

#### 2.5.3 Biot number and mass transfer coefficient

As a dimensionless parameter, the Biot number (*Bi*) is used to understand what the mass transfer and kinetic modeling express about the relative importance of internal diffusion resistance compared to external mass transfer resistance [22]. In fact, the *Bi* correlates with the relative transport resistances for the extraction of a solid phase in the liquid phase. The *Bi* is defined in Eq (16).

(16) $$\mathrm{Bi}=\;\frac{K_{T}.R}{D_{e}}$$

Where, $K_{T}$ (m/s) denotes the external mass transfer coefficient, R (m) is the particle radius, and $D_{e}$ (m^2^/s) is the diffusion coefficient within particles governed by Fick’s second law as shown in Eq. (14). The value of “$K_{T}$” was then calculated by Eq. (17).

(17) $$\ln\frac{C_{s}}{C_{s}-C_{t}}=\;\frac{K_{T}}{L_{C}}t$$

where, $L_{C}$ represents characterstic length of the sphere, $C_{s}\;\mathrm{and}\;C_{t}$ represent saturation concentration and extractable phenolic compounds in the supernatant at time *t*, respectively. Putting it all together, we could calculate Bi value as desired.

### 2.6 Thermodynamic analysis

The thermodynamic analyses are useful for explaining the energy changes and spontaneity that characterize the UAE process [11]. The main thermodynamic parameters include Gibbs free energy ($\Delta G^{o})$, enthalpy ($\Delta H^{o}$), and entropy $\left(\Delta S^{o}\right)$, with $\Delta G^{o}$ represents the maximum useful work achievable in a thermodynamic system under constant temperature and pressure conditions [14]. This value has been known to be associated with enthalpy and entropy when estimating the energy available during chemical changes. The precise formula of $\Delta G^{o}$ is

(18) $$\Delta G^{o}=\Delta H^{o}-T.\Delta S^{o}$$

To find $\Delta G^{o},$ we also can use the Gibbs free energy isotherm.

(19) $$\Delta G^{o}=-RTlnK_{\mathrm{eq}}$$

And then the Van 't Hoff equation can be derived to calculate Δ H° and Δ S°*.*

(20) $$\ln K_{\mathrm{eq}}=-\;\frac{\Delta H^{o}}{\mathrm{RT}}+\frac{\Delta S^{o}}{R}$$

where, $K_{\mathrm{eq}}$ is the equilibrium constant and is known as the ratio of the extracted quantity of phytochemical to the remaining unextracted quantity. This requires a different equation.

(21) $$K_{\mathrm{eq}}=\;\frac{C_{s}}{C_{\max}-C_{s}}$$

where, *C_s_* is the phytochemical compounds extracted after 80 min of ultrasound extraction time at a temperature *T* (K), and *C_max_* is the phytochemical compounds extracted after a complete extraction using an optimized solvent mixture.

### 2.7 Effect of ultrasound on kinetic parameters between UAE and CSE

For the well-defined kinetic parameters of UAE, the conventional shaking extraction (CSE) was conducted to determine the effect of ultrasound on the intensification of solid–liquid extraction. The UAE was performed under the following optimized conditions: extraction temperature of 50 °C, ethanol concentration of 50 %, solvent-to-solid ratio of 20 mL/g, extraction time of 80 min, ultrasonic frequency of 37 kHz, and amplitude of 50 %. In the case of the conventional method, CSE was conducted under identical conditions but without ultrasonic assistance, where samples mixed with ethanol solution in a 250 mL beaker were agitated uniformly using a low-temperature incubator (Hipoint, Taiwan) at a shaking speed of 200 rpm. Pseudo-second-order model parameters, including *C_s_, k, D_e_, K_T_*_,_ were calculated from the time series (10-80 min).

### 2.8 Statistical analysis

All experiments were prepared three times, and results were expressed as mean ± standard error of the mean (n = 3). Statistically significant values were analyzed using one-way analysis of variance (ANOVA) and the Turkey-Kramer HSD test with IBM SPSS Statistics (Version 22.0, New York, USA). Differences were significant at *p <* 0.05. The coefficient of determination (R^2^), mean square error (MSE), and root mean square error (RMSE) (represented by Eqs. (22), (23) and (24)) are calculated to evaluate the better fitness of the kinetics model for the experimental data.

(22) $$R^{2}=1-\frac{\sum_{i=1}^{n}{\left(Y_{\mathrm{Exp}.i}-Y_{\mathrm{Cal}.i}\right)}^{2}}{\sum_{i=1}^{n}{\left(Y_{\mathrm{Exp}.i}-{\overset{¯}{Y}}_{\mathrm{Exp}.i}\right)}^{2}}$$

(23) $$\mathrm{MSE}=\frac{1}{n}\sum_{i=1}^{n}{\left(Y_{\mathrm{Exp}.i}-Y_{\mathrm{Cal}.i}\right)}^{2}$$

(24) $$\mathrm{RMSE}=\sqrt{\frac{\sum_{i=1}^{n}{\left(Y_{\mathrm{Exp}.i}-Y_{\mathrm{Cal}.i}\right)}^{2}}{n}}$$

where, $Y_{\mathrm{Exp}.i}$ is the experimental values, $Y_{\mathrm{Cal}.i}$ is the calculated values, and *n* is the number of experimental data.

## 3 Results and discussion

### 3.1 *Influence of extraction conditions on the recovery of BACs from* SGL

**Fig. 1:**
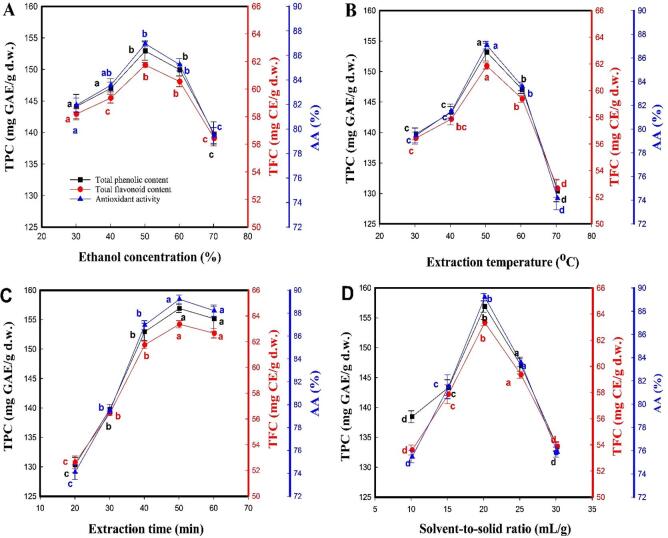
The effect of (A) ethanol concentration, (B) extraction temperature, (C) extraction time, (D) solvent-to-solid ratio on the TPC, TFC and AA in UAE from SGL. Different lowercase letters indicate a significant difference (*p* < 0.05) for TPC (black), TFC (red), and AA (blue) values.

The present study examined four distinct UAE process parameters, such as solvent, extraction time, temperature, and solvent-to-solid ratio. To observe the impact of each input variable, the single-factor experiment was set up with respect to the optimal result recorded from the highest combined values of TPC, TFC, and AA values. Solvent was frequently selected as the primary parameter to examine extraction yield because this variable is crucial to ensuring effective extraction. Unfortunately, no specific type of solvent exists for phenolic component extraction from SGL because of differences in chemical properties and polarity [23]. Therefore, a proper choice of solvent type and quantity was required to improve both efficiency and environmental effects. Ethanol-based solvents were selected in this study to recover BACs from SGL extract due to their demonstrated enhanced performance compared to other solvents and provided eco-friendly characteristics [19,24,25]. The effect of ethanol concentration on TPC, TFC, and AA from SGL was examined by varying ethanol concentration from 30 to 70 % (v/v), at temperature of 50 °C, extraction time of 40 min, and solvent-to-solid ratio of 20 mL/g. As shown in Fig. 1A. The TPC, TFC, and AA consistently increased with increasing ethanol concentration from 30 % to 50 %, reaching maximum yields of 152.92 ± 1.47 mg GAE/g d.w., 61.75 ± 0.18 mg CE/g d.w., and 86.95 ± 0.24 %, respectively. However, the curve behaves differently beyond 50 % of ethanol concentration; these values at 70 % ethanol concentration were significantly decreased by 8.6 % to 139.77 ± 1.91 mg GAE/g d.w., 56.44 ± 0.46 mg CE/g d.w., and 79.47 ± 0.65 %. This pattern demonstrates the suitability of a 50 % ethanolic solvent for extraction, which can be attributed to its balanced affinity for polar and semi-polar phenolic compounds. Ethanol solution promoted rapid dissolution, while water likely enhanced pore expansion for diffusion, resulting in a synergistic benefit of the ethanol–water mixture and yielding high extraction capacity. This result aligned with the UAE research on phenolic compounds derived from *Sargassum carpophyllum* [26] and Tunisian *Zizyphus lotus* fruits [27], which reported that the optimum operating condition included 50 % ethanol concentration for achieving high content of phenolic compounds.

The following analysis in this work was carried out to determine the effect of temperature on TPC, TFC, and AA of ethanolic extracts. Herein, the temperature varied from 30 to 70 °C in combination with fixed parameters at ethanol concentration of 50 %, extraction time of 40 min, and solvent-to-solid ratio of 20 mL/g_._ As depicted in Fig. 1B, the TPC yield increased from 139.77 ± 1.00 mg GAE/g d.w. at 30 °C to a peak of 153.18 ± 1.47 mg GAE/g d.w. at 50 °C, with TFC and AA reaching 61.85 ± 0.23 mg CE/g d.w. and 87.09 ± 0.31 %, respectively (*p* < 0.05). All responses then considerably decreased to 130.43 ± 1.78 mg GAE/g d.w., 52.67 ± 0.68 mg CE/g d.w., and 74.16 ± 0.96 % at 70 °C. The findings demonstrate that a temperature of 50 °C provided optimal conditions for the efficiency of extraction and antioxidant capacity of phenolic extracts. This temperature-dependent behavior occurred because increased extraction temperature from 30 to 50 °C initially enhanced solubility and mass transfer while reducing solvent viscosity and surface tension, thereby disrupting cell walls and promoting solvent entrance into plant matrices. Conversely, beyond the optimal threshold at higher temperatures, thermal degradation of phytochemical components caused the observed reduction. Moreover, the parallel trends observed among TPC, TFC, and AA throughout the temperature range are based on the hydrogen-donating ability of BACs, which overall enhanced the antioxidant capacity. These findings were also found in the research of Giri et al. (2024) [11], Pusty et al. (2024) [28], and Liao et al. (2021) [29], which confirmed the optimal temperature at around 50 °C for recovering the highest content of BACs from persimmon peel, red cabbage, and peanut shells, respectively.

The assistance of ultrasound in the extraction process mainly contributed to reduced processing time, energy, and cost savings. Therefore, the impact of extraction time was performed from 20 to 60 min during the UAE process under specific parameters at ethanol concentration of 50 %, temperature of 50 °C and solvent-to-solid ratio of 20 mL/g, with results depicted in Fig. 1C. In this figure, TPC consistently rose from 130.33 ± 1.27 mg GAE/g d.w. at 20 min to a maximum of 156.89 ± 0.69 mg GAE/g d.w. at 50 min, accompanied by TFC and AA values of 63.35 ± 0.28 mg CE/g d.w. and 89.20 ± 0.38 %, respectively (*p* < 0.05). The percentage increase at 50 min relative to 20 min was approximately 20.37 % for TPC, TFC, and AA. At 60 min, all responses exhibited marginal decline: TPC to 155.14 ± 2.12 mg GAE/g d.w., TFC to 62.65 ± 0.35 mg CE/g d.w., and AA to 88.21 ± 0.50 %. However, statistical analysis revealed that these differences between 50 and 60 min were not significant (*p* > 0.05); consequently, 50 min was selected as the optimal extraction time. The matched patterns in TPC, TFC, and AA values highlighted a robust correlation between TPC, TFC, and AA. The positive effect of extraction time was also observed in the UAE process for polyphenols derived from spruce bark (*Picea abies*) [30] and lycopene from guava fruit (*Psidium guajava L.)* [31], with optimum extraction times determined to be 40 and 33 min, respectively. Indeed, these findings are consistent with the principle that proper extraction time is essential for maximizing bioactive compound recovery while avoiding degradation due to prolonged ultrasonic exposure.

The impact of solvent-to-solid ratio was investigated on the yield of phenolics/flavonoids and antioxidant capacity of SGL extracts by conducting experiments at ethanol concentration of 50 %, temperature of 50 °C and extraction time of 50 min, as illustrated in Fig. 1D. TPC values consistently rose from 138.46 ± 0.99 mg GAE/g d.w. at 10 mL/g to a peak of 156.89 ± 0.69 mg GAE/g d.w. at 20 mL/g, with TFC and AA reaching 63.35 ± 0.24 mg CE/g d.w. and 89.20 ± 0.32 %, respectively. Statistical analysis revealed that these values at 20 mL/g exhibited significant differences (*p* < 0.05). At 30 mL/g, TPC, TFC, and AA displayed a considerable reduction to 133.46 ± 0.80 mg GAE/g d.w., 53.89 ± 0.30 mg CE/g d.w., and 75.88 ± 0.43 %. This decline can be attributed to excessive solvent diluting the concentration gradient, leading to reduced extraction efficiency [29,32]. These findings are in accordance with Bhagya Raj et al. (2020) [22], who reported an optimal ratio of 25:1 mL/g. It was noteworthy that higher solvent-to-solid ratios are generally recommended to achieve desirable results; the proper choice of solvent-to-solid ratio enhances both recovery performance and environmental sustainability.

### 3.2 Kinetic model of TPC extraction

**Fig. 2:**
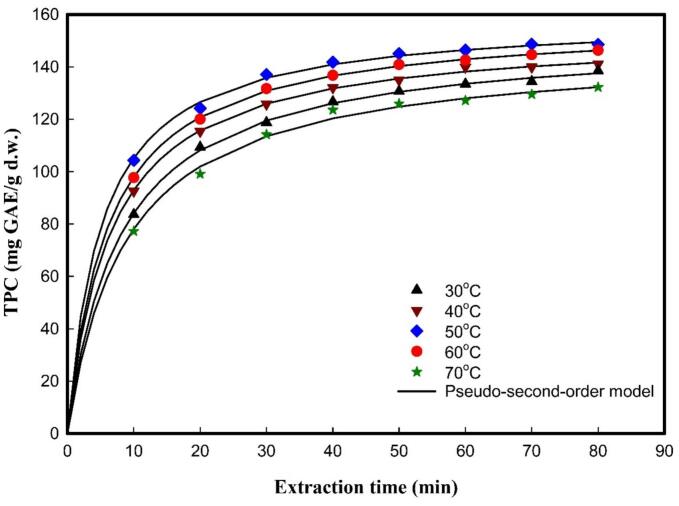
The effect of different temperatures on TPC extraction from SGL fitted to the pseudo-second-order model. The extraction was performed at extraction temperature of 50 °C, ethanol concentration of 50 %, solvent-to-solid ratio of 20 mL/g, and ultrasonic frequency of 37 kHz with 50 % amplitude.

The UAE kinetics for TPC recovery from SGL extract were modeled using a pseudo-second-order kinetic model. This kinetic model determined saturation concentration (*C_s_*) and extraction rate (*h*) of phenolic compounds under various temperatures from 30 to 70 °C and ultrasonic times from 10 to 80 min. Precisely, the TPC extraction response was measured at 10 min intervals, while other parameters fixed at ultrasonic frequency of 37 kHz with amplitude of 50 %, ethanol concentration of 50 %, and solvent-to-solid ratio of 20 mL/g. By using the data from Table S1, the pseudo-second-order model parameters were graphically fitted via linearized plots, as shown in Fig. 2. Then the saturation concentration (*C_s_*) and rate constant (*k*) were respectively obtained from the slopes (1/*C_s_*) and intercepts (1/*h*), where *h* = *kC_s_*^2^, by plotting *t*/*C_t_* against *t*. By looking at the flow in Fig. 2, the *C_s_* results showed a distinct temperature-dependent pattern. In the initial extraction stage, TPC consistently increased with rising temperature from 151.30 ± 1.21 mg GAE/g d.w. at 30 °C to an optimal value of 159.15 ± 0.94 mg GAE/g d.w. at 50 °C, then marginally declined to 154.69 ± 1.10 mg GAE/g d.w. at 70 °C. The highest concentration of *C_s_* was achieved at 50 °C in the extract with 80 min sonication, with marginal increases (< 2 %) after 60 min. This variability could be because of the possible effect of temperature on enhanced solvent penetration and cellular disruption at moderate temperatures, while higher temperatures may lead to thermal degradation that damages phenolic compound stability [33].

Similarly, the rate constant (*k*), which denotes the rate of extraction, followed a comparable nonlinear response pattern. In Table 1, the *k* × 10^−4^ values increased from 8.2876 ± 0.0943 g d.w./mg GAE min^−1^ at 30 °C to a peak of 12.3074 ± 0.5916 g d.w./mg GAE min^−1^ at 50 °C. Subsequently, the rate constant decreased to 9.8862 ± 0.4524 g d.w./mg GAE min at 70 °C. The decrease in rate constant should be caused by thermal degradation, which leads to a decrease in TPC. Higher rate constant values indicate a faster extraction of solute into the solvent, while higher saturation concentration values suggest that the maximum amounts of solute have been extracted from the sample [11]. The pseudo-second-order rate model successfully described the kinetic behavior of UAE for the phenolic compounds. To validate this kinetic approach, based on regression model evaluation metrics (R^2^, MSE, and RMSE), the pseudo-second-order kinetic equation was found to fit the observed phenolic content of the UAE extract, with R^2^ higher than 0.9936, and MSE and RMSE less than 1.4908, 1.2210, respectively. These statistical parameters confirmed the reliability of the kinetic model in describing the extraction behavior under the examined conditions. Together, the parameters *C_s_* and *k* indicate that 50 °C was the optimal temperature for maximizing both saturation concentration and extraction rate, in line with the biphasic extraction profile characterized by a rapid initial release during the washing phase, followed by slower diffusion (consistent with Fick’s second law). Similar observations have also been made by Bhagya Raj et al. (2020) [22], who also examined kinetic parameters for TPC extraction and found that higher temperatures also increase extraction rates at initial stages, but higher temperatures with prolonged ultrasonic time proved detrimental to phenolic compounds.

**Table 1:** Effect of temperature on pseudo-second-order model parameters (*C_s_* and *k*) for UAE of TPC from SGL.

T (^o^C)	*C_s_* (mg GAE/g d.w.)	*k* × 10^−4^ (g d. w. /mg GAE min)	R^2^	MSE	RMSE
30	151.30 ± 1.21^c^	8.2876 ± 0.0943^c^	0.9977	0.6738	0.8209
40	153.11 ± 0.90^bc^	10.1101 ± 0.1876^b^	0.9980	0.4964	0.7046
50	159.15 ± 0.94^a^	12.3074 ± 0.5916^a^	0.9936	1.3395	1.1574
60	157.31 ± 0.36^a^	10.5152 ± 0.1213^b^	0.9991	0.2519	0.4693
70	154.69 ± 1.10^b^	9.8862 ± 0.4524^b^	0.9941	1.4908	1.2210

The extraction was performed at extraction temperature of 50 °C, ethanol concentration of 50 %, solvent-to-solid ratio of 20 mL/g, extraction time of 80 min and ultrasonic frequency of 37 kHz with 50 % amplitude. The Tukey–Kramer HSD test was applied to compare means for *Cs* and *k* at different temperature. Data are expressed as mean ± SD. Means followed by different letters are significantly different at *p* < 0.05.

### 3.3 Diffusion coefficient and mass transfer coefficient

**Fig. 3:**
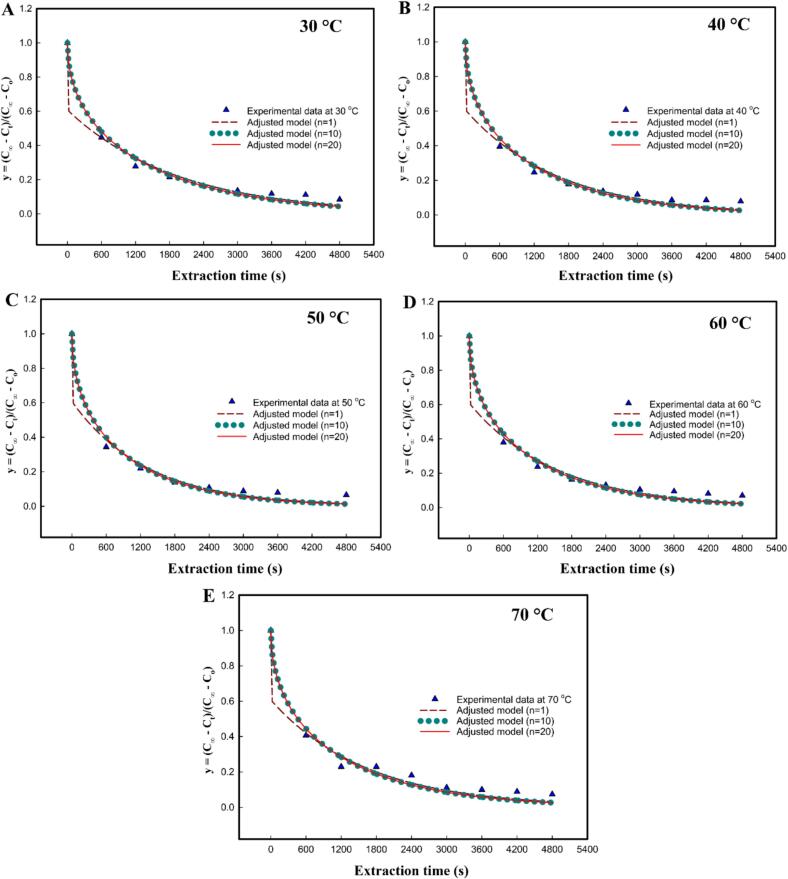
Fickian diffusion model fitting exprimental extraction kinetics using different series terms (n = 1, 10, 20) at temperatures: (A) 30 °C, (B) 40 °C, (C) 50 °C, (D) 60 °C, (E) 70 °C.

**Fig. 4:**
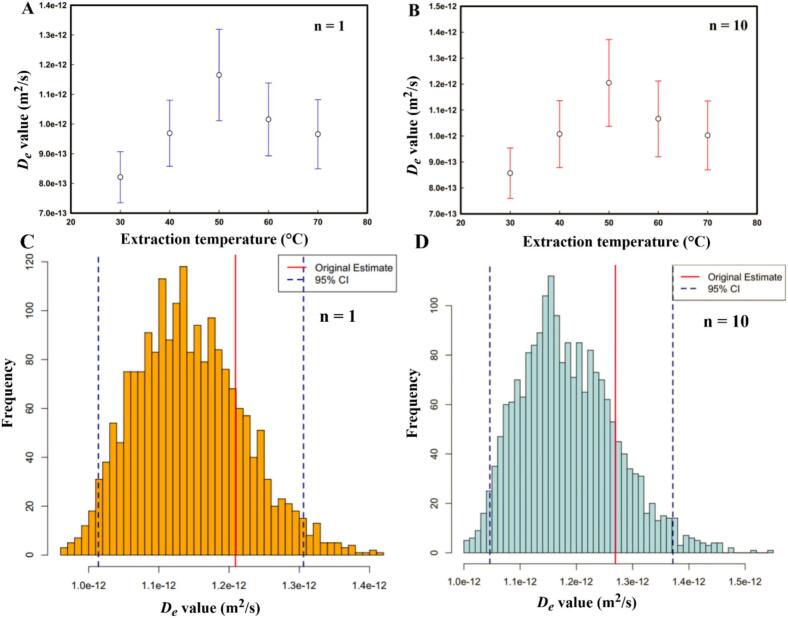
Bootstrap analysis of effective diffusivity: temperature dependence (A, B) and distribution at 50 °C (C, D) for 1-term and 10-term series solutions.

In solid–liquid extraction, the effective diffusion coefficient (*D_e_*) is a useful kinetic parameter for reflecting the diffusion rate of phenolic compounds from the SGL matrix into the ethanol/water solvent. From experimental data, the *D_e_* was calculated using Crank’s model by establishing the nonlinear least-squares regression. To evaluate the effect of series truncation on model accuracy, the analysis was done by using 1, 10, and 20 terms of the solution series to plot $y=\left(C_{\infty }-C_{t}\right)/\left(C_{\infty }-C_{0}\right)$ against *t* (s), as shown in Fig. 3**.** The resulting *D_e_* values within its statistical metrics were then derived from five different levels of temperature, ranging from 30 to 70 °C, as presented in Table 2. The adjusted model with only one term (n = 1) showed significant deviation, while the models with n = 10 and n = 20 were nearly coincident with negligible deviation. This indicates that with just 1 term, the model was not well adjusted, resulting in high variability. Further, as shown in Table 2, at each temperature tested, the *D_e_* calculated using the truncated 1-term series was also consistently lower than the value derived from the mathematically stable 10-term series. Moreover, the 95 % bootstrap confidence intervals for the n = 1 model show significant overlap between adjacent temperatures. These findings indicate that the number of terms in analytical solutions in series significantly affects the variability of experimental results. Therefore, 10 or more terms of the solution series are proposed here to use for obtaining *D_e_* of phenolic compounds from SGL. When using n = 10 terms, the *D_e_* values ranged from 8.728 to 12.690 × 10^−13^ m^2^/s, while temperature varied from 30 to 70 °C. Initially, *D_e_* was measured at 8.728 × 10^−13^ m^2^/s at the temperature of 30 °C, indicating the slowest diffusion kinetics. Subsequently, when temperature elevation initially enhanced diffusion efficiency, *D_e_* increased systematically from 8.728 × 10^−13^ m^2^/s at 30 °C to 10.410 × 10^-13^ m^2^/s at 40 °C by 19.27 % and peaked at 12.690 × 10^−13^ m^2^/s at 50 °C. These results also demonstrated that temperature factors significantly influence the binary diffusion coefficient (consistent with Fick’s second law), and then the targeted compound’s diffusion process could be divided into a fast diffusion phase and a low diffusion phase under each extraction temperature examined. This enhancement can be attributed to improved molecular mobility and decreased solvent viscosity under moderate thermal conditions. However, further temperature increases caused *D_e_* to decline considerably to 11.050 × 10^−13^ m^2^/s at 60 °C by 12.92 %, and it continued decreasing to 10.340 × 10^−13^ m^2^/s at 70 °C by a further 5.60 %. High temperatures may also induce solute degradation, thereby reducing the effective concentration gradient for diffusion. Based on the bootstrap analysis, the 95 % confidence interval (CI) for 30 °C (7.594 × 10^−13^– 9.539 × 10^−13^ m^2^/s) does not overlap with the 95 % confidence interval for 50 °C (10.370 × 10^−13^– 13.720 × 10^−13^ m^2^/s). This lack of overlap provides robust statistical evidence that the *D_e_* increase significantly with temperature ranging from 30 to 50 °C. Together, these findings indicate 50 °C as the optimal extraction temperature for maximizing phenolic recovery in this system (Fig. 4). Obviously, the observed increase in *D_e_* with temperature aligns with the increase in the pseudo-second-order rate constant (*k*); however, this process is typically limited by internal diffusion within the solid [34]. Therefore, determining the effective diffusion coefficient, alongside other kinetic parameters, is important for predicting extraction rates and optimizing industrial operating conditions to maximize target compound yield. The obtained *D_e_* values are within the range reported in literature for similar extraction processes. The next approach currently under investigation involves characterizing the mass transfer coefficient (*K_T_*) of TPC extraction. Following a similar vein, the *K_T_* values exhibited variation with temperature across from 30 to 70 °C, achieving an optimum value of 5.8335 ± 0.0112 at 50 °C. Furthermore, at any given temperature, the *K_T_* was greater than the *D_e_*, suggesting that external mass transfer resistance might be less critical than internal diffusion resistance in certain UAE processes. It is noteworthy that internal diffusion within the solid matrix could be a more critical rate-limiting step [11].

**Table 2:** Comparison of the number of terms in initial diffusivity estimate and statistical parameters for TPC response from SGL.

T (^o^C)	n = 1		n = 10		n = 20	
	*D_e_* x 10^−13^ (m^2^/s) ^(*)^	95 % CI x 10^−13 (**)^	*D_e_* x 10^−13^ (m^2^/s)	95 % CI x 10^−13 (**)^	*D_e_* x 10^−13^ (m^2^/s)	95 % CI x 10^−13 (**)^
30	8.256 ± 0.442	7.349 – 9.072	8.728 ± 0.496	7.594 – 9.539	8.728 ± 0.496	7.594 – 9.539
40	9.872 ± 0.583	8.576 – 10.800	10.410 ± 0.752	8.786 – 11.360	10.410 ± 0.752	8.786 – 11.360
50	12.090 ± 0.801	10.110 – 13.190	12.690 ± 0.854	10.370 – 13.720	12.690 ± 0.854	10.370 – 13.720
60	10.480 ± 0.636	8.928 – 11.380	11.050 ± 0.728	9.200 – 12.120	11.050 ± 0.728	9.200 – 12.120
70	9.808 ± 0.608	8.492 – 10.820	10.340 ± 0.680	8.694 – 11.350	10.340 ± 0.680	8.694 – 11.350

(*) SE, Bootstrap standard error; (**) 95 % Bootstrap confidence interval for *D_e_*.

### 3.4 Biot number

The Biot number (*Bi*) is known as a dimensionless value that quantifies the balance between two types of resistances to mass transfer during extraction. Calculation of the *Bi* makes it possible to identify the rate-limiting resistance, indicating whether internal diffusion or external mass transfer controls the extraction kinetics. Based on literature, when *Bi* > 50, mass transfer is mainly controlled by internal diffusion mechanisms, representing internal mass transfer as the rate-limiting step. Conversely, when *Bi* < 50, the rate-determining step is external mass transfer, meaning that the process is limited by diffusion from the solid surface to the bulk solvent [19]. As shown in Table 3, the *Bi* values were calculated within the range of 1146.79 to 1553.11 under different temperatures. Precisely, all examined temperatures yielded *Bi* values extremely higher than 50, with an average of 1349.95 ± 203.16, confirming that the rate-limiting step was internal mass transfer. This finding indicates that the UAE of SGL in the current study was limited by the relative transport resistances of phenolic compounds from the interior of the SGL to their surfaces. Furthermore, this result is also linked to the synergistic effects of acoustic cavitation, fluid turbulence, and solvent penetration in SGL extraction, as it effectively accelerates the transport of phytochemical from the inside of the solid to the surface. Rather, the result illustrates that the influence of temperature on the *Bi* values can vary with respect to the specific compounds and matrices used for the extraction process.

**Table 3:** Diffusion coefficient, mass transfer coefficient, Biot number, and thermodynamic parameters for TPC response.

T (^o^C)	*D_e_* x 10^−13^ (m^2^/s)(*)	*K_T_* × 10^−6^ (m/s)	Biot number	ΔH^o^ (kJ/mol)	ΔS^o^ (J/mol. K)	ΔG^o^ (kJ/mol)
30	8.728 ± 0.496^c^	5.4194 ± 0.0144^e^	1553.11 ± 3.00^a^	3.8151	32.8216	−6.1299
40	10.410 ± 0.752^bc^	5.6242 ± 0.0178^c^	1352.15 ± 3.28^b^			−6.4581
50	12.690 ± 0.854^a^	5.8335 ± 0.0112^a^	1146.79 ± 2.63^c^			−6.7864
60	11.050 ± 0.728^ab^	5.6953 ± 0.0103^b^	1286.04 ± 2.72^d^			−7.1146
70	10.340 ± 0.680^bc^	5.5571 ± 0.0016^d^	1354.08 ± 9.09^b^			−7.4428

The Tukey–Kramer HSD test was applied to compare means for *De*, *K_T_* and Bi at different temperature. (*) SE, Bootstrap standard error. The *K_T_* and Bi values are expressed as mean ± SD. Means followed by different letters are significantly different at *p* < 0.05.

### 3.5 Thermodynamic parameters for the UAE responses

In general, thermodynamic parameters provide insight into changes in energy, entropy, and spontaneity of a process [15]. For the present UAE system, the thermodynamic parameters, consisting of enthalpy change (ΔH^o^), entropy change (ΔS^o^), and Gibbs free energy change (ΔG^o^), are shown in Table 3. The calculated values of ΔH^o^ (3.8151 kJ/mol) and ΔS^o^ (32.8216 J/mol.K) were positive, indicating that the UAE process is endothermic. This suggests that the process relies on external energy input to proceed. The underlying rationale behind this observation is the mechanical energy generated by ultrasonic cavitation effectively overcomes this enthalpy barrier without the requirement for excessively high temperatures. Moreover, the relatively low ΔH^o^ value indicates that the process does not demand much energy input, thereby making UAE more efficient than conventional high-temperature extraction methods. Using Eq. (24), ΔG^o^ values can be calculated from ΔS^o^ and ΔH^o^ values over a temperature series. As can be seen in Table 2, all ΔG^o^ values, ranging from -6.1299 to -7.4427, were less than 0, demonstrating that the present UAE process is spontaneous at all tested temperatures. Additionally, the ΔG^o^ values also become increasingly more negative with increasing temperature, which confirms that UAE is practical and more efficient at higher temperatures. These results support optimizing UAE at high temperatures to achieve the highest TPC while proving that ultrasound increases thermodynamic efficiency. Taken together, these findings provide scientific insight into how ultrasound alters both kinetics and thermodynamics, thereby leading to higher efficiency. These conclusions are also in line with the research of Singh et al. (2025) [14], who reported that the ultrasound-microwave-assisted extraction approach for extracting BACs from pomegranate fruit peel using ethanol solvent exhibited endothermic, irreversible, and spontaneous properties. Their study yielded ΔG^o^, ΔH^o^, and ΔS^o^ for TPC of 8.127 to 10.068 kJ/mol, 11.588 kJ/mol, and 65.017 J/mol.K, respectively [14]. Also, other observations have been made from the UAE of dragon fruit peel, where the thermodynamic parameters demonstrated positive change in enthalpy (23.605) and entropy (102.171) for TPC, illustrating an increase in disorder during UAE. Moreover, the ΔG^o^ values ranged from −7.353 to −10.418, further supporting the thermodynamically spontaneous traits of the extraction process [22].

### 3.6 Comparative evaluation of mass transfer coefficients for TPC

**Fig. 5:**
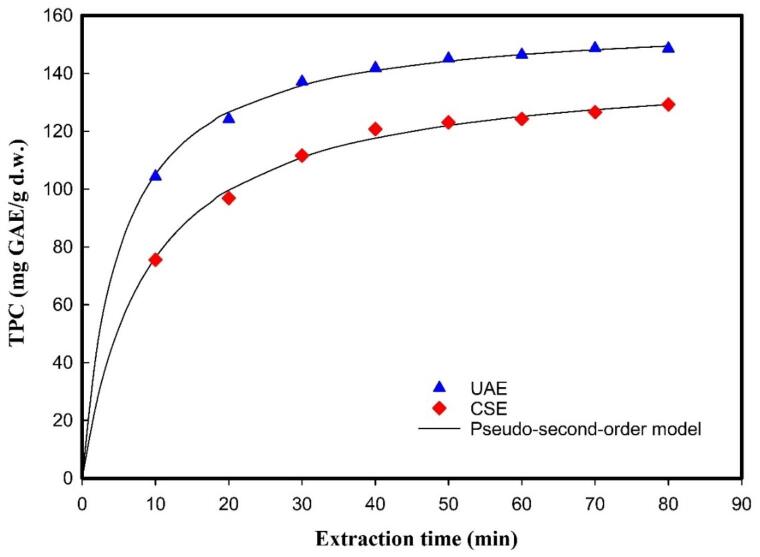
Effect of ultrasound on the TPC extraction from strawberry guava leaves and fitting to the pseudo-second-order model.

In order to evaluate the performance of ultrasound on the solid–liquid extraction. Based on the pseudo-second-order model, the kinetic parameters of phenolic compounds during UAE were identified and compared with those of conventional shaking extraction (CSE). The former utilized ultrasonic treatment at 37 kHz frequency and 50 % amplitude, while the latter relied solely on mechanical agitation. For both extraction systems, samples were analyzed in a time series ranging from 10 to 80 min, as shown in Fig. 5. Other fixed extraction parameters were maintained at the same conditions. The extraction yields during different extraction times were shown in Table S2. Based on the result, the value of *C_s_, k, D_e_*, and *K_T_* of both UAE and CSE were shown in Table 4. More precisely, UAE showed remarkably higher *C_s_* of 159.0087 ± 0.9572 mg GAE/g d.w., *k* of 12.3074 × 10^−4^ g d.w./mg GAE min, *D_e_* of 12.690 × 10^−13^ m^2^/s, and *K_T_* of 5.8335 × 10^−6^ m/s, as compared to CSE values of 143.4529 ± 1.044 mg GAE/g d.w., 7.9421 × 10^−4^ g d.w./mg GAE min, 7.613 × 10^−13^ m^2^/s, and 5.2058 × 10^−6^ m^2^/s, respectively. As previously discussed, the *C_s_* value relates to the equilibrium stage of the extraction process, representing the maximum concentration of solute that can be extracted from the solid matrix; a higher *C_s_* value represents that UAE achieves higher extraction yields. In the pseudo-second-order model, the *k* value is associated with the extraction kinetic rate, indicating the rate at which the solute reaches equilibrium. A higher *k* value indicates that the UAE creates a faster rate of extraction than CSE and a shorter time to reach equilibrium. Similarly, for the value of *K_T_* in both methods, a similar increased pattern was also observed. *K_T_* generally relates to external convective transfer at the solid–liquid interface, mainly controlled by turbulence, boundary layer thickness, and Biot number. The upward trend in *K_T_* of the UAE indicates enhanced external mass transfer, which leads to an overall rise in mass transfer, especially in the initial phase.

**Table 4:** Effect of ultrasound on pseudo-second-order model parameters (*C_s_, k, D_e_, K_T_*) for UAE and conventional shaking extraction (CSE) of TPC from SGL.

Parameters	UAE	CSE
*C_s_* (mg GAE/g d.w.)	159.0087 ± 0.9572^a^	143.4529 ± 1.044^b^
*k* × 10^−4^ (g d. w. /mg GAE min)	12.3074 ± 0.5916^a^	7.9421 ± 0.4630^b^
*D_e_* × 10^−13^ (m^2^/s)	12.690 ± 0.854^a^	7.613 ± 0.374^b^
*K_T_* × 10^−6^ (m/s)	5.8335 ± 0.0112^a^	5.2058 ± 0.0587^b^
R^2^	0.9936	0.9911
MSE	1.3395	2.6673
RMSE	1.1574	1.6332

The Tukey–Kramer HSD test was applied to compare means for all parameters of UAE and CSE. Data are expressed as mean ± SD. Means followed by different letters are significantly different at *p* < 0.05.

## 4 Conclusions

In this study, the mechanism of ultrasound-assisted phenolic extraction from *Psidium cattleianum leaves* was successfully unveiled through kinetic modeling and thermodynamic parameters. The single-factor experimental design revealed optimal extraction conditions at 50 % (v/v) ethanol concentration, 50 °C extraction temperature, 50 min extraction time, and 20 mL/g solvent-to-solid ratio, yielding TPC, TFC, and AA of 156.89 ± 0.69 mg GAE/g d.w, 63.35 ± 0.24 mg CE/g d.w., 89.20 ± 0.32 %, respectively. Pseudo-second-order kinetic analysis showed a temperature-dependent UAE process with two distinct stages: a rapid initial release, followed by a slower diffusion stage. Ultrasound enhanced mass transfer through cavitation mechanisms, with optimal *D_e_* and *K_T_* values recorded at 50 °C. Thermodynamic analysis confirmed an endothermic, entropic, and spontaneous process that was feasible at higher temperatures. Furthermore, UAE demonstrated superior performance compared to CSE in saturation concentrations, rate constants, and mass transfer parameters. It is noteworthy that this study offers a reference framework for the application of ultrasound in extraction as a green technology for valorizing SGL waste into phenolic-rich extracts with detailed mechanistic insights. For future research, this work suggests developing robust optimization of the UAE process for industrial continuous reactor systems based on simulation research integrated with a kinetics model for real-time control, which aligns with circular economy principles by minimizing solvent and energy consumption.

## CRediT authorship contribution statement

**Hoang Duy Huynh:** Writing – original draft, Methodology, Investigation, Formal analysis, Data curation. **Parushi Nargotra:** Validation. **Hui-Min David Wang:** Resources. **Yung-Hsiang Tsai:** Validation. **Chien-Chih Chiu:** Resources. **Chwen-Jen Shieh:** Conceptualization. **Yung-Chuan Liu:** Writing – review & editing. **Chia-Hung Kuo:** Writing – review & editing, Visualization, Supervision, Conceptualization.

## Funding

This work was supported by research funding grants provided by the National Science and Technology Council of Taiwan (NSTC 111-2221-E- 992-005-MY3).

## Declaration of competing interest

The authors declare that they have no known competing financial interests or personal relationships that could have appeared to influence the work reported in this paper.
