# Supplementary material for: Unveiling the mechanism of ultrasound-assisted phenolic extraction from Psidium cattleianum leaves: Kinetic, mass transfer, and thermodynamic insights
Source: Ultrason Sonochem. 2025 Nov 11;123:107675. doi: 10.1016/j.ultsonch.2025.107675 (PMC12664415; doi:10.1016/j.ultsonch.2025.107675)
Supplement: Supplementary Data 1 [file mmc1.docx]

# Unveiling the mechanism of ultrasound-assisted phenolic extraction from *Psidium cattleianum* leaves: Kinetic, mass transfer, and thermodynamic insights

Hoang Duy Huynh^abc^, Parushi Nargotra^a^, Hui-Min David Wang^d^, Yung-Hsiang Tsai^a^, Chien-Chih Chiu^e^, Chwen-Jen Shieh^f^, Yung-Chuan Liu^g,^* Chia-Hung Kuo^ab,^*

^a^ Department of Seafood Science, National Kaohsiung University of Science and Technology, Kaohsiung 811, Taiwan

^b^ Institute of Aquatic Science and Technology, National Kaohsiung University of Science and Technology, Kaohsiung 811, Taiwan

^c^ Faculty of Applied Technology, Yersin University of Dalat, Dalat 670000, Vietnam

^d^ Graduate Institute of Biomedical Engineering, National Chung Hsing University, Taichung 402, Taiwan

^e^ Department of Biotechnology, Kaohsiung Medical University, Kaohsiung 807, Taiwan

^f^ Biotechnology Center, National Chung Hsing University, Taichung 402, Taiwan

^g^ Department of Chemical Engineering, National Chung Hsing University, Taichung 402, Taiwan

*****Correspondence: Dr. Yung-Chuan Liu (telephone: +886-4-22853769, fax: +886-4-22854734, email: ycliu@dragon.nchu.edu.tw); Dr. Chia-Hung Kuo (telephone: +886-7-3617141 ext. 23646, fax: +886-7-3640634, e-mail: kuoch@nkust.edu.tw)

**Table S1.** Pseudo-second-order kinetics for TPC of SGL extracted at different temperatures.

| **Extraction time (min)** | **30 ^o^C** | | **40 ^o^C** | | **50 ^o^C** | | **60 ^o^C** | | **70 ^o^C** | |
| --- | --- | --- | --- | --- | --- | --- | --- | --- | --- | --- |
|  | **Exp.** | **Cal.** | **Exp.** | **Cal.** | **Exp.** | **Cal.** | **Exp.** | **Cal.** | **Exp.** | **Cal.** |
| 10 | 83.64 ± 0.51 | 84.17 ± 0.75 | 92.60 ± 0.94 | 93.01 ± 0.56 | 104.34 ± 1.35 | 105.19 ± 1.01 | 97.71 ± 0.43 | 98.04 ± 0.63 | 91.83 ± 1.74 | 93.49 ± 1.47 |
| 20 | 109.32 ± 1.50 | 108.16 ± 0.91 | 115.41 ± 0.57 | 115.72 ± 0.60 | 124.18 ± 1.35 | 126.62 ± 0.54 | 120.02 ± 0.91 | 120.79 ± 0.57 | 119.20 ± 1.17 | 116.54 ± 1.20 |
| 30 | 118.74 ± 0.56 | 119.52 ± 0.98 | 125.81 ± 1.36 | 125.97 ± 0.65 | 137.14 ± 0.18 | 135.84 ± 0.37 | 131.68 ± 0.82 | 130.92 ± 0.52 | 126.74 ± 1.84 | 126.98 ± 1.06 |
| 40 | 126.70 ± 1.71 | 126.15 ± 1.03 | 132.10 ± 1.05 | 131.81 ± 0.69 | 141.83 ± 1.10 | 140.97 ± 0.37 | 136.78 ± 1.28 | 136.65 ± 0.48 | 131.87 ± 0.87 | 132.93 ± 0.99 |
| 50 | 130.73 ± 1.01 | 130.48 ± 1.06 | 135.02 ± 0.71 | 135.58 ± 0.71 | 145.05 ± 0.61 | 144.25 ± 0.42 | 140.88 ± 0.66 | 140.34 ± 0.46 | 137.50 ± 1.35 | 136.78 ± 0.96 |
| 60 | 133.47 ± 1.65 | 133.55 ± 1.08 | 139.92 ± 0.90 | 138.22 ± 0.74 | 146.43 ± 1.18 | 146.51 ± 0.48 | 142.55 ± 0.71 | 142.91 ± 0.45 | 139.24 ± 1.09 | 139.47 ± 0.95 |
| 70 | 134.44 ± 0.99 | 135.82 ± 1.09 | 139.93 ± 0.52 | 140.17 ± 0.75 | 148.67 ± 1.28 | 148.18 ± 0.53 | 144.60 ± 0.50 | 144.80 ± 0.43 | 141.02 ± 1.20 | 141.46 ± 0.95 |
| 80 | 138.52 ± 1.23 | 137.58 ± 1.10 | 141.09 ± 1.29 | 141.66 ± 0.77 | 148.58 ± 0.81 | 149.45 ± 0.57 | 146.32 ± 0.46 | 146.26 ± 0.42 | 143.34 ± 0.97 | 142.98 ± 0.95 |

The extraction was performed at extraction temperature of 50 ^o^C, ethanol concentration of 50 %, solvent to solid ratio of 20 mL/g, and ultrasonic frequency of 37 kHz with 50 % amplitude.

**Table S2**. The effect of ultrasound-assisted extraction and conventional shaking extraction on TPC extracted from SGL.

| **Extraction time (min)** | **Ultrasound-assisted**  **extraction** | | **Conventional shaking**  **extraction** | |
| --- | --- | --- | --- | --- |
|  | **Exp.** | **Cal.** | **Exp.** | **Cal.** |
| 10 | 112.56 ± 0.86 | 114.56 ± 1.03 | 78.23 ± 1.07 | 79.31 ± 0.79 |
| 20 | 136.03 ± 1.04 | 137.97 ± 0.62 | 100.34 ± 1.00 | 103.22 ± 0.66 |
| 30 | 148.67 ± 0.97 | 148.05 ± 0.61 | 115.60 ± 0.68 | 114.76 ± 0.61 |
| 40 | 155.42 ± 1.07 | 153.67 ± 0.70 | 125.07 ± 0.82 | 121.55 ± 0.61 |
| 50 | 157.81 ± 0.71 | 157.25 ± 0.79 | 127.48 ± 1.12 | 126.02 ± 0.64 |
| 60 | 159.01 ± 0.42 | 159.73 ± 0.87 | 128.72 ± 0.81 | 129.19 ± 0.67 |
| 70 | 161.23 ± 1.25 | 161.56 ± 0.93 | 131.12 ± 1.29 | 131.56 ± 0.70 |
| 80 | 162.10 ± 0.80 | 162.95 ± 0.98 | 133.86 ± 0.69 | 133.39 ± 0.73 |

The extraction was performed at extraction temperature of 50 ^o^C, ethanol concentration of 50 %, solvent to solid ratio of 20 mL/g, and ultrasonic frequency of 37 kHz with 50 % amplitude
